# Integrated Multiomics Analyses Revealing Different Molecular Profiles Between Early- and Late-Stage Lung Adenocarcinoma

**DOI:** 10.3389/fonc.2021.746943

**Published:** 2021-10-21

**Authors:** Dongsheng Yue, Weiran Liu, Liuwei Gao, Lianmin Zhang, Tao Wang, Shanshan Xiao, Yingxue Fu, Nan Li, Rui Lin, Yao Hu, Lieming Ding, Zhenfa Zhang, Bin Zhang, Changli Wang

**Affiliations:** ^1^ Department of Lung Cancer, Lung Cancer Center, Tianjin Medical University Cancer Institute and Hospital, National Clinical Research Center for Cancer, Key Laboratory of Cancer Prevention and Therapy, Tianjin’s Clinical Research Center for Cancer, Tianjin, China; ^2^ Department of Anesthesiology, Tianjin Medical University Cancer Institute and Hospital, National Clinical Research Center for Cancer, Key Laboratory of Cancer Prevention and Therapy, Tianjin’s Clinical Research Center for Cancer, Tianjin, China; ^3^ Department of Enhanced Recovery After Surgery, Tianjin Medical University Cancer Institute and Hospital, National Clinical Research Center for Cancer, Key Laboratory of Cancer Prevention and Therapy, Tianjin, China; ^4^ Department of R&D, Hangzhou Repugene Technology Co., Ltd., Hangzhou, China; ^5^ Department of Medical, Betta Pharmaceutical Co., Ltd, Hangzhou, China

**Keywords:** lung adenocarcinoma, whole-genome sequencing, RNA-seq, ATAC-seq, integrated multi-omics analyses

## Abstract

The molecular differences in genetic and epigenetic profiling between early-stage (ES) and late-stage (LS) lung adenocarcinoma (LUAD), which might help to understand cancer progression and biomarker guided precision treatment, need further be investigated. In this study, we performed comprehensive analysis using multi-omics next-generation sequencing (NGS) on tissue samples from 7 ES (stage I) and 10 LS (stage III/IV) LUAD patients to study molecular characteristics between the two groups. Characterization of the genomic and transcriptomic profiles showed stage-specific somatic mutations, copy number variations (CNVs) and differentially expressed genes (DEGs). LS samples tend to have more TP53, ERBB2 and CHD4 mutations. Gene copy number loss occurs in immune-related gene pathways in the late stage of LUAD. ATAC-seq analysis showed that LS samples harbored more open chromatin peaks around promoter regions and transcription start sites (TSS) than ES samples. We then identified the known transcription factor (TF) binding motifs for the differentially abundant ATAC-seq peaks between the ES and LS samples and found distinct regulatory mechanisms related to each stage. Furthermore, integrative analysis of ATAC-seq with WGS and RNA-seq data showed that the degree of chromatin accessibility is related to copy number changes, and the open chromatin regions could directly regulate the expression of some DEGs. In conclusion, we performed a comprehensive multi-omics analysis of the early and late stages of LUAD and highlighted some important molecular differences in regulatory mechanisms during cancer progression. Those findings help to further understand mechanism and biomarker related targeted therapy.

## Introduction

Lung cancer is currently the leading cause of cancer-related mortality among malignant tumors worldwide (1). Lung adenocarcinoma (LUAD) accounts for 40% of all lung cancer cases and is the most common histological subtype of non-small cell lung cancer (NSCLC) (2). The high mortality rate observed in patients with lung cancer is correlated with the late tumor stage at diagnosis, which results in a lack of curative treatment and leads to a relatively worse prognosis and clinical outcome (3). Improved knowledge of the molecular characteristics between the early and late stages of LUAD could be crucial for understanding the development and progression of this cancer and developing patient-specific precision treatment. Previous studies, especially those conducted by The Cancer Genome Atlas (TCGA), have extensively characterized the molecular features of LUAD, such as genomic mutations, differential gene expression, and protein function alterations that lead to tumorigenesis and cancer progression (4, 5). Specifically, LUAD is usually associated with oncogenic driver mutations located in EGFR, KRAS and ALK, and approximately 65% of LUAD patients harbor at least one of these driver mutations (6). However, the mechanisms and regulatory networks identified by multi-omics data in the developmental process of LUAD remain largely unexplored.

Increasing data have indicated that epigenetic dysregulation is widespread and plays important roles in lung cancer initiation and progression (7). Assay for transposase-accessible chromatin using sequencing (ATAC-seq), an efficient and sensitive method for analysis of the integrative epigenome, can provide chromatin accessibility maps of the entire genome to identify gene regulatory regions (8–10). For instance, recent study uncovered distinct open chromatin patterns between LUAD and lung squamous cell carcinoma (LUSC) and regulatory networks linking open chromatin peaks to copy number variations (CNVs) and gene expression alterations by comprehensively analyzing open chromatin data with whole-genome sequencing (WGS) and RNA-seq data from 50 NSCLC patients (8). Nonetheless, few studies have focused on elucidating the molecular characteristics of the early and late stages of LUAD by multi-omics data to more thoroughly understand the gene regulatory mechanism during LUAD progression and development.

In the present study, we generated comprehensive multi-omics data by various next-generation sequencing (NGS) technologies, including targeted 474-gene NGS panels, WGS, RNA-seq and ATAC-seq datasets from 7 early-stage (ES, stage I) and 10 late-stage (LS, stage III/IV) LUAD patients, and performed an integrative analysis on these omics data. Briefly, we first characterized the genomic and transcriptomic profiles of the 17 patients and identified somatic mutations, CNVs, and differentially expressed genes (DEGs) specific to each group. We then identified open chromatin regions in each sample using ATAC-seq and annotated these open chromatin peaks to the nearest genes. Transcription factor (TF) binding motifs were identified using differentially abundant ATAC-seq peaks between the early and late stages to depict their different gene regulatory profiles. In addition, we conducted functional and gene network analysis for open chromatin peak-associated genes. Finally, we performed an integrative analysis of the ATAC-seq data with the WGS and RNA-seq data to identify key regulatory elements and elucidate how they are associated with gene regulatory networks in LUAD progression.

## Materials and Methods

### Patients and Clinical Information

This study enrolled 7 ES (stage IA) and 10 LS (stage III/IV) treatment-naïve LUAD patients. All 17 patients were nonsmokers. Tumor tissue and paired blood samples were obtained from each patient who underwent surgery or biopsy at Tianjin Medical University Cancer Institute and Hospital between September 2015 and April 2016. Staging was performed according to the 8th Edition of the International Staging of Thoracic Malignancies (3). The collection of all samples was approved by the Ethical Committees of Tianjin Medical University Cancer Hospital, and all participants provided written informed consent.

### Sample Processing and Sequencing

Resected tumor tissues were snap-frozen in liquid nitrogen and peripheral blood samples were collected before treatment from the 17 LUAD patients. The tumor cell percentage of each tissue was more than 80%, estimated by two pathologists on hematoxylin and eosin-stained slides (**Supplementary Figure S1**). DNA and total RNA were extracted from fresh frozen tissue using the DNeasy Blood & Tissue Kit (Qiagen NV, Venlo, Netherlands) and RNeasy Mini Kit (Qiagen NV, Venlo, Netherlands), respectively, following the manufacturer’s protocols. Peripheral blood samples were collected in cfDNA BCT tubes (Streck Laboratories, Omaha, NE), stored at 15–30°C and processed within 72 hours. Each tube was centrifuged at 1,600 g for 10 minutes at 4°C. Pellets containing peripheral blood lymphocytes were stored at -80°C before further use; supernatants were centrifuged at 16,000 g for another 10 minutes, aliquoted into sterile 1.5 ml tubes and stored at -80°C before extraction. Genomic DNA from peripheral blood lymphocytes was extracted using the QIAamp DNA Mini and Blood Mini Kit (Qiagen NV, Venlo, Netherlands) according to the manufacturer’s protocol. The Qubit 3.0 Fluorometer and Qubit dsDNA HS Assay kits (Life Technologies, Carlsbad, CA) were used to quantify DNA following the recommended protocol. The quality of extracted RNA was evaluated with a NanoDrop 2000 (Thermo Fisher) and Agilent 2100 Bioanalyzer (Agilent Technologies). The DNA/RNA libraries were constructed and described in the following sections. All libraries were sequenced on the HiSeq X-Ten platform (Illumina, San Diego, CA). Raw sequencing data were then processed by bcl2fastq to convert BCL files into FASTQ format. Sequencing reads were filtered with the criteria that each read should have less than 20% low-quality bases (phred score < 20) and have 2 or fewer undetermined bases (base N).

### Whole Genome Sequencing Data

The library was constructed using an Illumina TruSeq Nano DNA Low Throughput Library Prep Kit following the manufacturer’s instructions. Reads were mapped to the hg38 genome using bwa-0.7.15 (9). Indel realignment and base recalibration were performed using GATK-3.8 to improve indel detection sensitivity and correct base quality score bias (10). MuTect2 was used for single nucleotide variation (SNV) and insert-deletion (INDEL) calling. All variants were annotated using snpEff-2.3.7 (11). Tumor sample CNVs were called using Control-FreeC (version 9.5, parameters: ploidy = 2, breakPointThreshold = 0.5, coefficientOfVariation = 0.05, mateOrientation = FR) with paired blood samples as controls (12). GISTIC2 (version 2.0.23) was used to identify CNV regions that were significantly amplified or deleted across the ES or LS samples (parameters: -genegistic 1 -smallmem 1 -broad 1 -brlen 0.98 -conf 0.90 -savegene 1 -qvt 0.1) (13).

### Targeted Panel Sequencing Data

The library was constructed using the KAPA Hyper Prep kit (Kapa Biosystems) and hybridized with probes targeting all the exons of 474 cancer-related genes using the SureSelectXT Target Enrichment System (Agilent Technologies) following the manufacturer’s instructions. The 474 cancer-related genes were listed in **Supplementary Table S1**. The concentration and size of the prepared library were evaluated using a Qubit 3.0 Fluorometer (Life Technologies, Carlsbad, CA) and 2100 Bioanalyzer (Agilent Technologies). Read alignment and postprocessing were the same as those for the WGS data. Varscan-2.4.1 was used for SNV and INDEL calling (14). All variants were annotated using snpEff-2.3.7 (11). Only coding region variants (SNVs and INDELs) with variant allele frequency (VAF) ≥ 1% were kept for further analysis. For calculation of the tumor mutation burden (TMB), we applied the criterion from a previous publication (VAF ≥ 5% for tissue) (15).

### RNA Sequencing Data

The RNA-seq library was constructed using the NEBNext Ultra Directional RNA Library Prep Kit (New England Biolabs) following the manufacturer’s instructions and qualified using a Qubit 3.0 Fluorometer (Life Technologies, Carlsbad, CA) and 2100 Bioanalyzer (Agilent Technologies). Sequencing reads were mapped to the hg38 genome and Ensembl 93 genome annotation using STAR (16). Gene expression quantification was performed using RSEM (17) to obtain the fragment per kilo exon per million reads (FPKM) of each gene. DEGs were analyzed using DEseq2, and the clusterProfiler of the R software package was used to perform gene pathway enrichment analysis with a threshold of p < 0.05 (18–20). The enrichment analysis mapping was performed using a p-value.

### ATAC-Seq Data

ATAC-seq was performed as previously described with minor modifications (21, 22). Briefly, to prepare nuclei, 2~5 mg of frozen tissue was put into liquid nitrogen, and the nuclei were precipitated by grinding, lysis, centrifugation and washing after quick-freezing. Following the nuclei prep, the transposase reaction mix was added to the nuclei precipitate and the transposition reaction was incubated at 37°C for 1.5 h. Immediately after incubation, the sample was purified using the Qiagen MinElute PCR Purification Kit. The purified DNA was added to the PCR reaction solution for pre-amplification, and then the number of cycles corresponding to 1/4 of the maximum fluorescence position was determined as the number of cycles to be added to the PCR reaction for re-amplification of the pre-amplified PCR system. The amplified PCR products were purified using AMPure XP beads. The purified DNA libraries were quantified using a Qubit 3.0 Fluprometer and a dsDNA HS assay kit (Life Technologies, Carlsbad, CA, USA. Then the Agilent Bioanalyzer 2100 (Agilent) and High Sensitivity DNA Reagents (Agilent) were used for quality control of the library. The fragment sizes in the sequencing library approximately ranged from 250 bp to 1000 bp (**Supplementary Figure S2**). All libraries were sequenced on the Illumina HiSeq-X10 with 150 bp paired-end reads.

Clean reads were mapped to the hg38 genome using bwa-0.7.15 (9). Duplicate reads were removed using MarkDuplicates in the Picard package. TN5 read shift correction was performed following the ENCODE standard protocol (23). The *multiBamSummary* from deepTools 2.0 was used to compute the read coverages genome-wide for the ATAC-seq BAM files of all 17 samples. The resulting matrix was then used to calculate Spearman’s rank correlation coefficient between each pair of samples. MACS2 was used for peak calling from the mapped reads (24). The complexity of the library (PBC) and FRIP value were used for quality control. The differentially abundant open chromatin regions were identified using DiffBind. *De novo* and known motif enrichment analyses of the differentially abundant peak regions between the ES and LS samples were performed by the HOMER function *findMotifsGenome.pl*. The closest gene method was used to link a gene to an open chromatin region on which TF binding could affect the expression of the gene (25). To depict the relationship between the ATAC-seq peak and CNVs, we first intersected open chromatin peaks with somatic CNV regions in each sample and then investigated the relationship using the peak signal values and copy numbers.

## Results

### Clinical Characteristics of the Patients

To reveal the multi-omics differences between the early and late stages of LUAD, we analyzed tumor samples from seven ES (stage IA) and 10 LS (stage IIIA/IV) Chinese LUAD patients in this study (**Table 1** and **Supplementary Table S2**). All 17 patients were nonsmokers, and the median ages of the ES and LS groups were 59 and 50 years, respectively. Both groups had roughly equal numbers of male and female patients. WGS, targeted panel sequencing, RNA-seq and ATAC-seq were performed for all 17 tumor tissue samples, and paired blood samples were used as controls for the WGS and targeted panel sequencing.

**Table 1:** Clinical Characteristics of early and late stage of LUAD patients.

Characteristics	ES (Early Stage N = 7)	LS (Late Stage N = 10)
**Age-yrs.**		
Median	59	50
Range	48-67	33-62
**Sex**		
Male	4 (57%)	5 (50%)
Female	3 (43%)	5 (50%)
**Race**		
Asian	7 (100%)	10 (100%)
**Smoking status – no. (%)**		
Never	7 (100%)	10 (100%)
**Histologic type – no. (%)**		
Adenocarcinoma	7 (100%)	10 (100%)
**EGFR mutation type – no. (%)**		
Exon 19 deletion	3 (43%)	4 (40%)
L858R	3 (43%)	1 (10%)
No mutation	1 (14%)	5 (50%)
**Stage – no. (%)**		
IA	7 (100%)	0
IIIA	0	2 (20%)
IIIB	0	3 (30%)
IV	0	5 (50%)

EGFR, epidermal growth factor receptor; ES, Early Stage; LS, Late Stage.

### Genomic Variation Analysis for Early- and Late-Stage Lung Adenocarcinomas

To obtain a global landscape of genomic variations of the early and late stages of LUAD, we performed WGS of the 17 tumor samples. No significant difference was found between the number of somatic mutations in exon regions of the ES samples and that of the LS samples (Student’s t test, P-value = 0.93, **Supplementary Figure S3**). We further characterized the somatic CNVs for each tumor sample based on the WGS data and identified genes targeted by those genomic regions that were significantly amplified or deleted across the ES and LS samples (**Supplementary Figure S4** and **Table S3**). The results showed that 373 and 333 genes exhibited significant amplifications among the ES and LS patients, respectively (FDR q-value < 0.1), and 64.3% (240/373) of them overlapped between the two groups (**Supplementary Figure S5**). Functional enrichment analysis showed that these amplified genes from both the ES and LS groups were involved in “basal transcription factors”, “nucleotide excision repair”, “cell cycle”, “RNA transport” and “FoxO signaling pathway”, which are related to tumor cell proliferation (**Supplementary Figures S6A, C**). For genes located in deleted CNV regions, the ES and LS groups harbored a set of 7861 and 1092 genes, respectively, and had nearly no overlap (**Supplementary Figure S5**). Deleted genes in the ES group were enriched in “basal cell carcinoma”, “proteoglycans in cancer”, “biosynthesis of amino acids”, “renal cell carcinoma” and “mTOR signaling pathway” (**Supplementary Figure S6B**). In contrast, genes located in deletion regions among the LS patients were significantly enriched in immune-related processes, such as “RIG-I-like receptor signaling pathway”, “natural killer cell mediated cytotoxicity”, “Toll-like receptor signaling pathway”, “cytokine-cytokine receptor interaction” and “JAK-STAT signaling pathway”, indicating a functional deficiency of the immune system in the late stage of LUAD (**Supplementary Figure S6D**).

**Figure 1:**
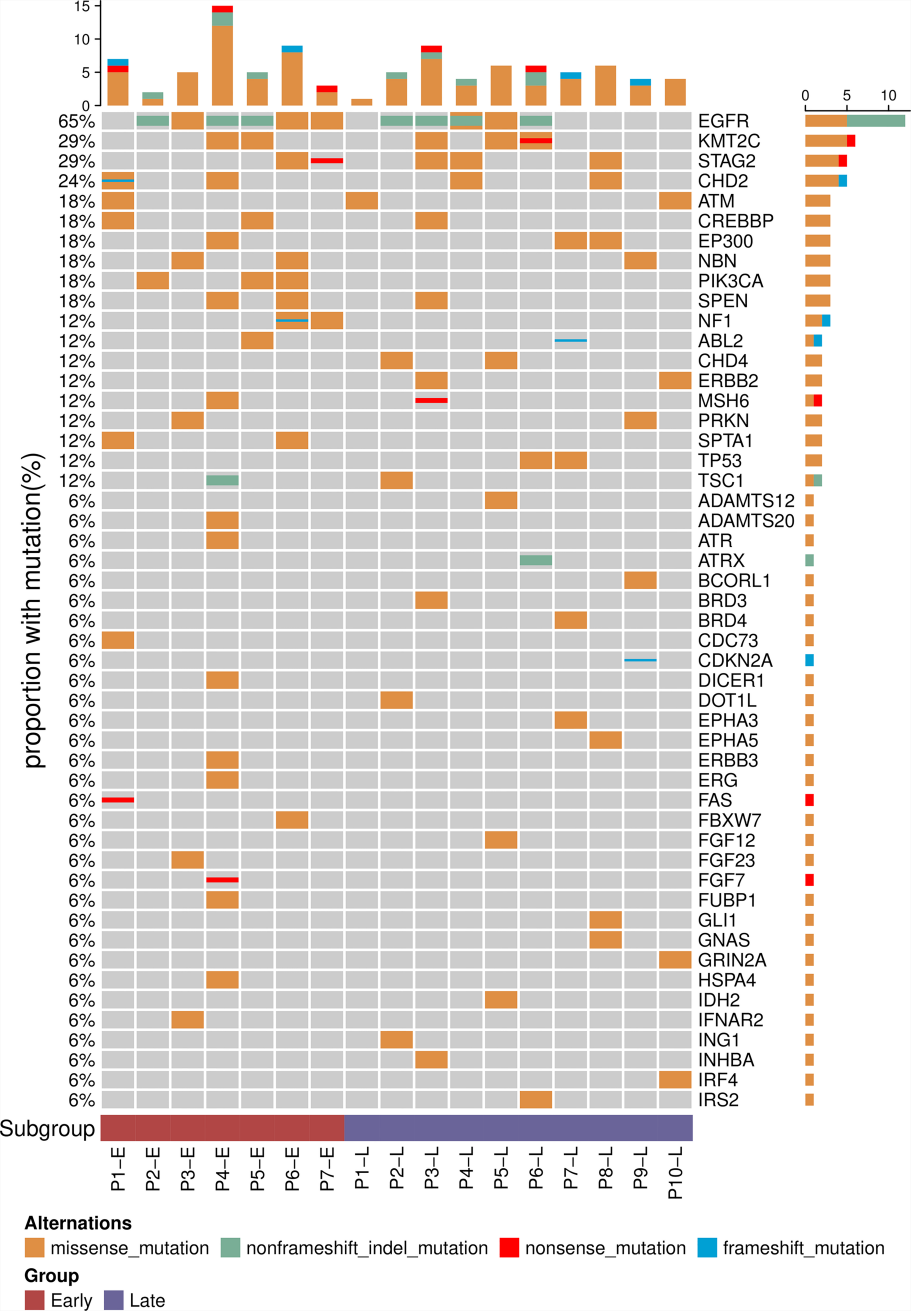
Mutational profiles of early- and late-stage LUAD. Top 50 genes with the highest mutation frequency among the 17 patients. The frequency of each gene mutated in the cohort is displayed on the left.

To more precisely characterize the mutation profiles of the ES and LS samples, we performed targeted NGS panel sequencing of cancer-related genes using a 474-gene panel (**Figure 1** and **Supplementary Figure S7**). As expected, EGFR mutations showed the highest rate (65%, 11 out of 17) in the 17 Chinese LUAD patients. The frequency of EGFR mutations showed no significant difference between the ES group and LS group, where 6 of the 7 samples in the ES group and 5 of the 10 samples in the LS group were found to have one of the EGFR-TKI-sensitive mutations (21L858R and 19del) (Fisher’s exact test, P-value = 0.16). In addition, mutations in PIK3CA (18%, 3 of 17) and NF1 (12%, 2 of 17) were observed only in the ES samples, while mutations in CHD4, ERBB2 and TP53 (all have a mutation rate of 12%) were found only in the LS group in this LUAD cohort. We analyzed the incidence of these gene mutations using a large cohort with 183 lung adenocarcinomas from Imielinski’s study (**Supplementary Table S4**) (26). PIK3CA, NF1, CHD4, ERBB2, and TP53 mutations were detected in both ES and LS patients. Consistent with our observation, CHD4, ERBB2 and TP53 mutations are more prevalent in LS patients.

### RNA-Seq Reveals Expression Differences Between Early- and Late-Stage Lung Adenocarcinomas

To characterize the expression differences between the ES and LS groups, we conducted RNA-seq analysis of the 17 tumor samples and identified 87 upregulated and 173 downregulated genes in the LS group compared to the ES group [absolute log_2_FC (fold change) > 2 and adjusted P-value < 0.05, **Supplementary Figure S8** and **Supplementary Table S5**]. For cancer driver genes, ERBB4 showed significantly lower expression levels in the LS samples. ALK and NTRK2 showed much greater variation in the LS samples than in the ES samples. We also identified a group of keratin family genes (KRT6A, KRT6B, KRT14, and KRT16) with higher expression levels in LS LUAD. Interestingly, the keratin expression level distinguishes the subgroups of other solid tumors (27–30) and is associated with poor clinical outcome (31). Although ERBB2, PIK3CA, and TP53 were not identified as DEGs, their expression variations in the LS samples were significantly larger than those in the ES samples, which could be due to changes in the regulatory network during the progression of tumors. Forty-seven noncoding RNAs (ncRNAs) were also differentially expressed between the ES and LS groups. Among them, MIR31HG, DRAIC, LUCAT1, and LINC00261 were previously identified as prognostic markers. Two other ncRNAs, VLDLR-AS1 and LINC01919, were highly expressed in the ES samples. Survival analysis of the 17 patients showed that the higher expression of VLDLR-AS1 and LINC01919 was associated with better overall survival (**Supplementary Figures S9** and **S10**). TCGA_LUAD database was used to validate the finding. High expression of VLDLR-AS1 was correlated with better prognosis (P=0.017), but LINC01919 was not (P=0.19) (**Supplementary Figures S11** and **S12**). Some studies reported that VLDLR-AS1 was associated with the prognosis of thymoma (32, 33). Thus, VLDLR-AS1 may serve as potential prognostic markers for LUAD.

**Figure 2:**
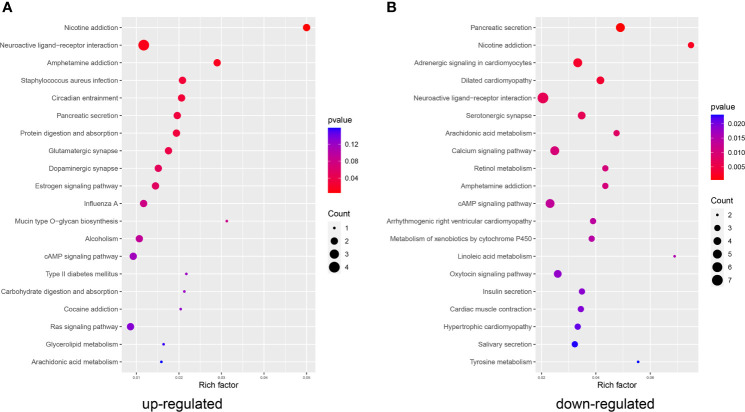
KEGG analysis. Functions and pathways enriched in the **(A)** upregulated and **(B)** downregulated genes in the LS group compared to the ES group.

Kyoto Encyclopedia of Genes and Genomes (KEGG) pathway enrichment analysis of these DEGs was performed to determine the functional or pathway differences between the early and late stages of LUAD. We found that genes upregulated in the LS group were mainly enriched in “protein digestion and absorption”, “Circadian entrainment” and “RAS signaling pathway” (**Figure 2A**). KRAS-driven lung cancers represent an aggressive form of NSCLC (34). The downregulated genes in the LS group were enriched in several important signaling pathways, including the “calcium signaling pathway” and “cAMP signaling pathway” (**Figure 2B**). cAMP was shown to modulate DNA damage and repair to regulate cellular survival and affect the prognosis of lung cancer patients (35). In addition, altered expression of specific Ca^2+^ channels and Ca^2+^-binding proteins can contribute to tumorigenesis and tumor growth in lung cancer (36).

### ATAC-Seq Identifies Differences in Chromatin Accessibility Between Early- and Late-Stage Lung Adenocarcinomas

**Figure 3:**
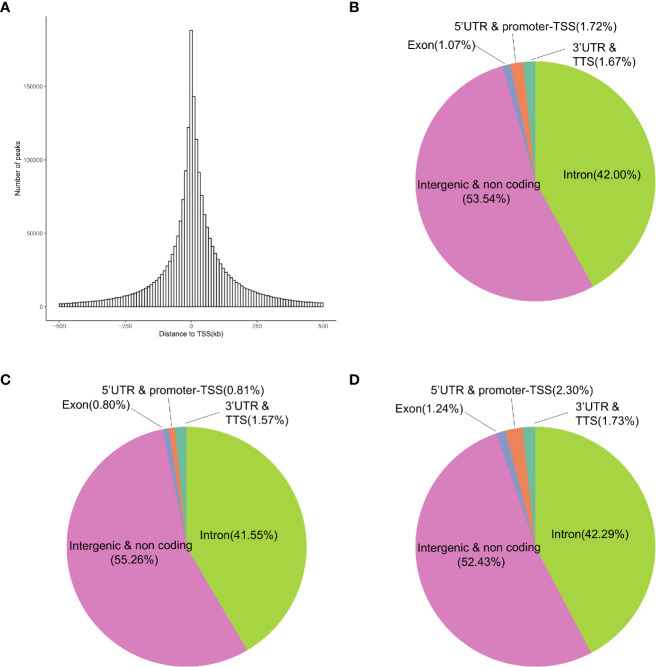
Genomic annotation of open chromatin peaks in the ES and LS samples. **(A)** Distribution of the distances of peaks to the TSS of the nearest genes. **(B–D)** Pie charts showing the distribution of peaks in different genomic regions in all 17 samples **(B)**, the ES group **(C)** and the LS group **(D)**.

The differential gene expression between the ES and LS groups may be related to chromatin accessibility-associated gene regulation. We used ATAC-seq, which evaluates genome-wide chromatin accessibility (21), to investigate the epigenetic differences between the ES and LS groups. The numbers of open chromatin peaks in the ES and LS samples ranged from 67,272 to 142,369 and 81,152 to 152,937, respectively (**Supplementary Figure S13A**). Hierarchical clustering of the 17 samples based on the ATAC-seq data showed two clusters corresponding to the samples in the ES and LS groups (**Supplementary Figure S13B**), suggesting that ATAC-seq profiles could serve as a good classification index of the early and late stages of LUAD. We further investigated the genomic distribution of ATAC-seq open chromatin peaks (**Figure 3**). An average of 1.72% of the ATAC-seq peaks were located close to the transcription start site (TSS) (**Figures 3A, B**). The majority of ATAC-seq peaks (95.54%) were located in the intergenic and noncoding regions and the intron region, suggesting that the ATAC-seq peak regions were likely to affect gene expression through remote regulatory elements (**Figure 3B**). Only 0.81% of the ATAC-seq peaks were located at the 5’-untranslated region (UTR) and promoter-TSS region for the ES group of LUAD patients (**Figure 3C**), but samples from the LS group had 2.3% ATAC-seq peaks at the same region (**Figure 3D**), a nearly 3-fold increase (chi-squared test, P-value < 2.2e-16). These results indicate that LS LUAD shows greater transcriptional activation than ES LUAD.

**Figure 4:**
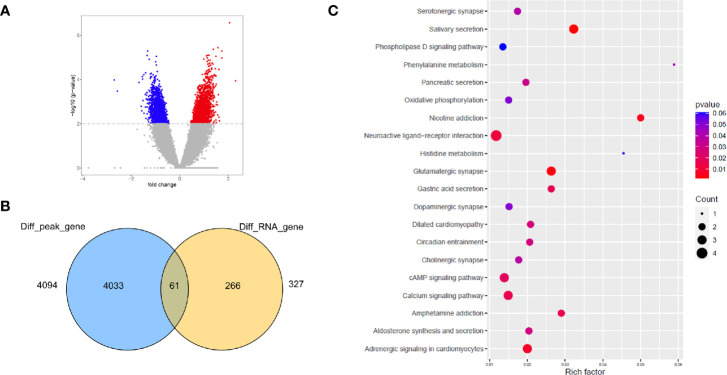
Integrative analysis of open chromatin peaks with DEGs detected by RNA-seq. **(A)** Volcano plot shows the ATAC-seq peaks specific to the ES (blue) and LS (red) samples. **(B)** Venn diagram showing the number of differentially abundant ATAC-seq peak-associated genes and DEGs. **(C)** KEGG pathway enrichment analysis of the overlapping genes of ATAC-seq and RNA-seq.

Open chromatin may affect RNA expression through TFs that bind to DNA sequence motifs known as TF binding sites (TFBSs) (37). We further performed differential peak analysis and identified 5860 differential open chromatin regions (P-value < 0.01) between the ES and LS groups (**Figure 4A** and **Supplementary Table S6**). Motif analysis of these differential ATAC-seq peak regions revealed 4 and 6 known TFBS motifs for the ES and LS groups, respectively (**Table 2**). For instance, the binding site motif for Cdx2 was enriched in the open chromatin regions of the ES group but not the LS group. Cdx2 is a TF that is critical in the developmental process of many multicellular organisms. Cdx2 was shown to cooperate with other proteins to inhibit LUAD metastasis (38). In contrast, the TFBS motif of MYB was identified in the ATAC-seq peaks for the LS group but not the ES group. MYB is a highly conserved TF that is widely expressed in proliferating cells and plays an important role in the cell cycle. MYB is also upregulated and promotes NSCLC growth and migration (39). Additionally, the binding site for the TF NeuroD1 was enriched in the ES group. NeuroD1 is a member of the NeuroD family of TFs and activates the transcription of genes containing DNA sequences known as E-boxes. NeuroD1 participates in the development of neural lineages and neural differentiation (40). Recently, NeuroD1 was also shown to promote malignant behaviors in neuroendocrine lung cancers and to target the Myc oncogene (41). These results indicate that the open chromatin regions for Cdx2 TFBSs may be involved in inhibiting cell metastasis and that NeuroD1 may promote tumorigenesis in ES LUAD, while MYB may contribute to tumor progression in LS LUAD.

**Table 2:** Motif analysis of the ATAC-seq peaks in ES and LS group of LUAD patients.

Stage	Motif name	Motif logo	P-value
Early	Cdx2	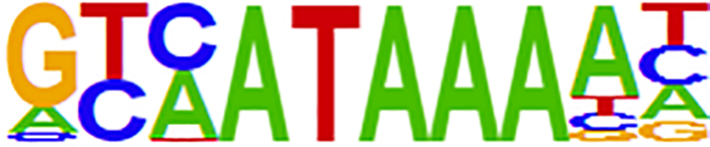	1e-2
	NeuroD1	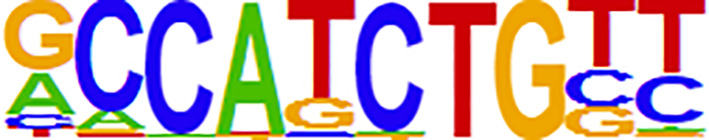	1e-2
	Pitx1: Ebox	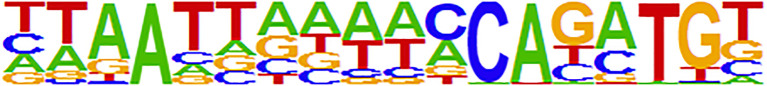	1e-2
	Pax7	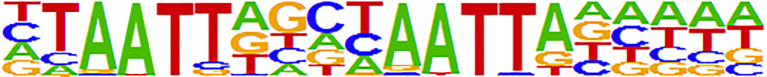	1e-2
Late	AMYB	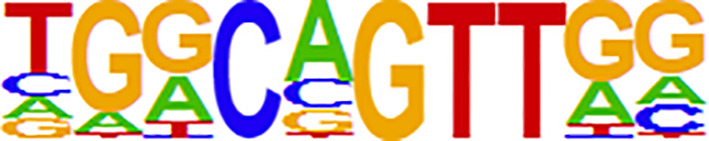	1e-3
	MYB	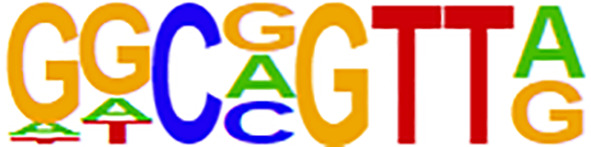	1e-2
	IRF3	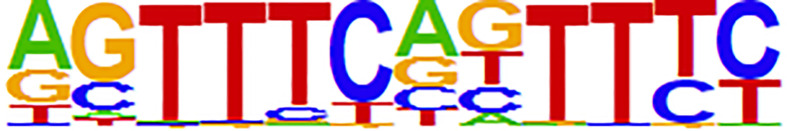	1e-2
	PBX1	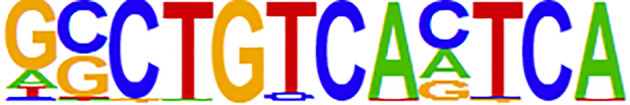	1e-2
	BMYB	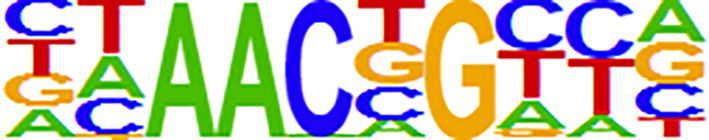	1e-2
	Pbx3	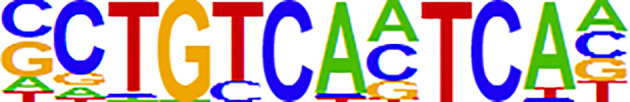	1e-2

ES, Early Stage; LS, Late Stage; Cdx2, caudal type homeobox 2; NeuroD1, neuronal differentiation 1; Pitx1:Ebox, paired like homeodomain 1: Enhancer-box; Pax7, paired box 7; AMYB, MYB proto-oncogene like 1; MYB, MYB proto-oncogene, transcription factor; IRF3, interferon regulatory factor 3; PBX1, PBX homeobox 1; BMYB, MYB proto-oncogene like 2; Pbx3, PBX homeobox 3.

### Integrative Analysis of ATAC-Seq Peaks With Copy Number Variation and Differential Gene Expression

To test whether open chromatin peaks are associated with somatic CNVs in the early and late stages of LUAD, we intersected open chromatin peaks with somatic CNV regions in each sample. The results showed that ATAC-seq peaks located in somatic CNV regions that had high copy numbers (CN > 6) tended to have stronger ATAC-seq peak signal intensity than those located in regions with low copy numbers (3≤ CN ≤6) in both the ES and LS samples (except P10-L) (**Supplementary Figure S14A**), which is consistent with a previous study reporting a similar trend in NSCLC patients (8). Interestingly, we found that in CNV gain regions, the correlation coefficient between the copy number and ATAC-seq peak signal among the LS samples was higher than that in the ES samples, suggesting that different genomic structure variations between early and late stages resulted in different degrees of chromatin accessibility (**Supplementary Figure S14B**).

To further determine whether the ATAC-seq data correlated with differential expression of genes, we evaluated the overlap between differentially abundant open chromatin region-related genes and DEGs. We identified 61 overlapping genes based on the ATAC-seq and RNA-seq data (**Figure 4B**); these genes are mainly involved in ligand-receptor binding and several signaling pathways (**Figure 4C**). Strikingly, many functional pathways involved in the nervous system/synapse were enriched in the 61 overlapping genes; these pathways included neuroactive ligand-receptor interactions, serotonergic synapses, cholinergic synapses, dopaminergic synapses, and glutamatergic synapses (**Figure 4C**). Consistently, many of these functional groups were also identified in the DEGs revealed by RNA-seq and peak regions in the ATAC-seq data and included neuroactive ligand-receptor interactions and glutamatergic synapses. Indeed, studies have revealed many genes involved in functions in both the nervous system and cancers. For instance, neurotrophic receptor tyrosine kinase (NTRK) is an important driver for many cancer types, including NSCLC, colorectal cancer, bladder cancer, and glioma (42). Moreover, NTRK family members have essential functions in the nervous system by regulating axon and dendrite growth, ion channels and neurotransmitter receptors, and synaptic strength and plasticity (43). However, the nerve axon guidance factor SLIT2 promotes tumorigenesis, angiogenesis and metastasis in many cancer types, including pancreatic cancer (44, 45). These results suggest that the genes that are involved in the normal functions of nervous system could drive tumorigenesis if ectopically expressed or dysregulated in other tissues (46).

### Functional and Network Analysis of Open Chromatin Region-Associated Genes

**Figure 5:**
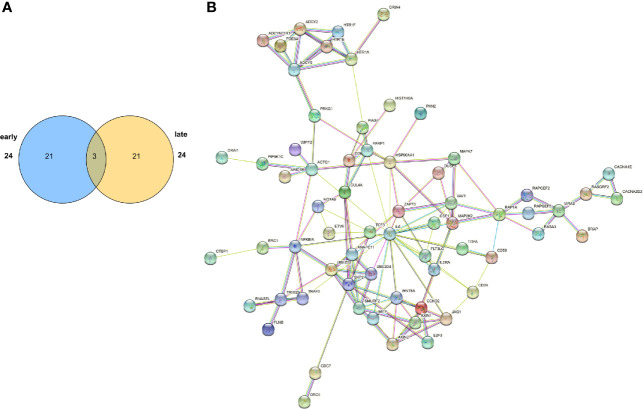
Functional and network analysis of differentially abundant open chromatin peaks between the ES and LS groups. **(A)** Venn plot showing the number of pathways significantly enriched in the ES- or LS-specific ATAC-seq peak-associated genes. **(B)** STRING network analysis of pathways enriched in the LS-specific ATAC-seq peak-associated genes.

The gene closest to the differentially abundant open chromatin regions is likely affected by the change in the regulatory region. Further characterization of the differentially abundant ATAC-seq peaks between the ES and LS groups (absolute fold change > 1 and P-value < 0.05) identified 1009 ATAC-seq peak-associated genes, including 319 ES-related and 690 LS-related genes. The most significantly dysregulated peak-associated genes were checkpoint kinase 2 pseudogene 2 (CHEK2P2) and long intergenic nonprotein coding RNA 1031 (LINC01031) in the ES group and Homo sapiens RNA, 5S ribosomal 1 (RNA5S1) and mitochondrial ribosomal protein L23 (MRPL23) in the LS group. KEGG pathway analysis of these ATAC-seq peak-associated genes showed that among the top 24 pathways enriched in the ES and LS groups, only 3 were shared by both groups (**Figure 5A** and **Supplementary Figure S15**), suggesting major differences between the two groups in epigenetic regulation. The identified pathways participate in a wide range of biological functions, including signaling pathways, tumor-related gene functions, neurological functions, secretion, and metabolism. For instance, genes in the ES group were enriched in the ErbB and phosphatidylinositol signaling pathways, whereas those in the LS group were enriched in the RAS signaling and cell cycle pathways. Interestingly, RAS signaling was also found to be enriched in upregulated genes in the LS group in our RNA-seq analysis (**Figure 2B**). These results indicate the importance of signal transduction pathways in the developmental stages of LUAD. In addition, our analysis found that several groups of neuro-related genes, such as axon guidance, glutamatergic synapses, and neuroactive ligand-receptor interactions, were related to potential regulatory differences in the early and late stages of LUAD. Although the relationship between neuro-related genes and cancers has not been fully elucidated, a recent study found that axon guidance genes are also related to tumorigenesis and metastasis (44).

We further performed protein-protein interaction (PPI) network analysis for the ATAC-seq peak-associated genes in the ES and LS groups (**Figure 5B** and **Supplementary Figure S13C**). In the ES PPI network, PIK3R1, CDH2, EGF, GJA1 and GLRA2 had the most connections to other genes, while in the late stage, SKP2, HSP90AA1, IL6, MAP2K2 and ZAP70 were hub genes in the network. Among them, PIK3R1 has been reported to act as a tumor suppressor (47). IL6/STAT3 signaling plays an important role in premetastatic niche formation in the lung (48). Activated MEK2, i.e., MAP2K2, could transform epithelial cells and induce high-grade adenocarcinomas in a mouse orthotopic transplantation model (49). Taken together, functional and network analysis of ATAC-seq peak-associated genes demonstrated that the early and late stages of LUAD displayed distinct epigenetic regulatory landscapes.

## Discussion

The molecular features of LUAD, such as genomic mutations, gene expression, and epigenetic regulation, have been extensively characterized in previous studies (4, 5). However, few studies have systematically investigated multi-omics differences, especially chromatin accessibility changes, between the early and late stages of LUAD. In this study, we generated and analyzed the ATAC-seq profiles of the tumor tissues of 7 ES (stage IA) and 10 LS (stage IIIA-IV) LUAD patients, along with their WGS, targeted panel sequencing and RNA sequencing data. Both WGS and targeted NGS panel sequencing identified different mutation patterns between the ES and LS groups. Importantly, we found that a large portion of the CNV gain regions among the ES samples were identical to those in the LS samples, while the CNV loss profiles between the two groups were largely different. Further, we observed that the ATAC-seq profiles could classify ES and LS LUAD samples, suggesting that chromatin accessibility among LUAD patients may serve as a novel type of molecular marker. Based on open chromatin peaks in the samples, we identified ES- and LS-specific clusters and further analyzed the changes in chromatin accessibility from the early to late stages of LUAD. Moreover, we found that the LS group tended to have more open chromatin regions near the promoter-TSS region than the ES group, indicating higher transcriptional activity in LS LUAD.

To characterize the regulatory roles of ES and LS-specific open chromatin regions, we identified the TFBS motifs enriched in each group. This exploration uncovered different transcriptional regulators that dominated in the early and late stages of LUAD. In addition to the expected enrichment for binding sites of TFs already known to play critical roles in LUAD, we identified several TFs whose binding site motifs were specifically enriched in the ES or LS group. For instance, we found that the NeuroD1 motif was significantly enriched in open chromatin regions of the ES group, indicating the involvement of neuro-related functions in the development of cancers (44). The differentially abundant open chromatin peaks between the ES and LS groups could also be associated with key genes related to tumor development. We identified numerous genes that were associated with ES and LS-selective ATAC-seq peaks and found that these genes were indeed enriched in known LUAD-related genes, which suggested that ATAC-seq peaks could potentially be utilized to identify key genes in LUAD.

Network analysis of the ATAC-seq peak-associated genes offered a way to investigate the relative importance of these genes in the context of biological functions. We found that the hub genes (PIK3R1, IL-6, and MAP2K2) in the PPI network composed of open chromatin region-associated genes all have known associations with tumor development, indicating that the gene expression regulation of these genes at the chromatin level may contribute to the progression from the early to late stage of LUAD. Importantly, through the integration of ATAC-seq and transcriptome data, we further investigated those genes that were dysregulated in both the ATAC-seq and RNA-seq profiles. The combined analysis of ATAC-seq and RNA-seq data provided more precise information on the effect of potential regulatory differences from differentially abundant open chromatin regions. The enriched pathways of these overlapping genes were closely related to neuroconduction and synapse-related functions, which further proved the connection between neuro-related factors and tumor development (46).

We compared the overlap between DEGs, somatic mutation, CNVs and differential OCPs (**Supplementary Table S7**). For example, the expression of BRDT was upregulated in LS patients, BRDT-related CNV deletion occurred in ES patients, and there was no difference in OCP signal between ES and LS patients. Therefore, the differential expression of BRDT may be caused by CNV. FBN2, which is upregulated in LS patients, showed no difference in CNV between ES and LS patients, but the OCP signal was stronger in LS patients. Therefore, the upregulation of FBN2 in LS patients is likely to be driven by OCP. NTRK2 was upregulated in LS patients, NTRK2-related CNV deletion occurred in LS patients, and OCP signal was weaker in LS patients. It is possible that the high expression of NTRK2 in LS patients is due to the trans-regulation of other OCPs that are not overlap.

Our study has several limitations. First, the sample size was relatively small. However, as the number of patients in the ES and LS groups was comparable, we identified some significant differences in the chromatin regulatory landscape between ES and LS LUAD. Moreover, a recent study that analyzed the open chromatin changes in 50 primary NSCLC cases demonstrated that the subgroup of NSCLC patients with fewer open chromatin regions was significantly associated with early tumor stage (8), which is consistent with our finding that the ES samples had fewer ATAC-seq peaks in the promoter-TSS region than the LS samples. Another limitation of our study is that we did not rule out disturbances from other nontumor cells, such as infiltrating immune cells and stromal cells. Future studies with a larger number of cases and normal cell types as controls will be needed to further confirm our findings.

In summary, we generated multi-omics profiles including ATAC-seq, WGS, and RNA-seq data from tumor tissues of ES and LS LUAD patients and performed an integrative analysis to elucidate the complicated regulatory network during the development of LUAD. Our study provides a novel perspective for understanding the changes in chromatin accessibility from the early to late stages of LUAD and offers a unique resource that may be manipulated for developing potential therapies for LUAD in the future.

## Data Availability Statement

The original contributions presented in the study are included in the article/**Supplementary Material**. Further inquiries can be directed to the corresponding authors.

## Ethics Statement

The studies involving human participants were reviewed and approved by The Ethical Committees of Tianjin Medical University Cancer Hospital. The patients/participants provided their written informed consent to participate in this study.

## Author Contributions

CW and BZ were responsible for conception and design. DY, WL, and LG performed development of methodology. DY, WL, LG, LZ, and LD contributed to acquisition and quality control of data. CW, BZ, DY, WL, LG, LZ, TW, SX, YF, YH, and LD contributed to analysis and interpretation of data. DY, BZ, WL, YF, NL, and LG drafted the manuscript. BZ and CW contributed to review and revision of the manuscript. CW, ZZ, and BZ contributed to study supervision. All authors contributed to the article and approved the submitted version.

## Funding

This work was supported by the National Natural Science Foundation of China (No. 81772484).

## Conflict of Interest

TW, SX, YF, NL, RL, and YH are employed by Hangzhou Repugene Technology Co., Ltd. LD is employed by Betta Pharmaceuticals Co., Ltd.

The remaining authors declare that the research was conducted in the absence of any commercial or financial relationships that could be construed as a potential conflict of interest.

## Publisher’s Note

All claims expressed in this article are solely those of the authors and do not necessarily represent those of their affiliated organizations, or those of the publisher, the editors and the reviewers. Any product that may be evaluated in this article, or claim that may be made by its manufacturer, is not guaranteed or endorsed by the publisher.

## Supplementary Material

Supplementary Figure S1 Representative HE staining images of ES and LS tissues (×4 magnification; the lower right corner, ×40 magnification).

Supplementary Figure S2 The fragment sizes in the sequencing library approximately ranging from 250 bp to 1000 bp.

Supplementary Figure S3 The number of somatic mutations in exon regions in ES and LS samples.

Supplementary Figure S4 Line chart shows significant copy number gains (red) and losses (blue; FDR < 0.1) in ES and LS LUAD.

Supplementary Figure S5 Venn diagram of genes targeted by amplified or deleted CNV regions among ES and LS samples.

Supplementary Figure S6 Functional enrichment of genes targeted by amplified (left) or deleted (right) CNV regions among ES (top) and LS (bottom) samples. The enriched signaling pathways of amplified genes in ES **(A)** and LS samples **(C)**. The enriched signaling pathways of deleted genes in ES **(B)** and LS samples **(D)**.

Supplementary Figure S7 The complete mutation profile of ES and LS LUAD.

Supplementary Figure S8 RNA-seq data analysis. **(A)** Volcano plot shows the differentially expressed genes (DEGs) between ES and LS groups. **(B)** Heatmap shows the hierarchical clustering of ES and LS samples based on the expression profiles of DEGs.

Supplementary Figure S9 Kaplan–Meier estimates among 17 patients evaluated for expression of ncRNA VLDLR-AS1.

Supplementary Figure S10 Kaplan–Meier estimates among 17 patients evaluated for expression of ncRNA LINC01919.

Supplementary Figure S11 Kaplan–Meier estimates based on TGCA_LUAD database evaluated for expression of ncRNA VLDLR-AS1.

Supplementary Figure S12 Kaplan–Meier estimates based on TGCA_LUAD database evaluated for expression of ncRNA LINC01919.

Supplementary Figure S13 ATAC-seq data analysis. **(A)** Bar plot shows the number of open chromatin peaks in the 17 LUAD samples. **(B)** Hierarchical clustering of the Spearman correlation of the ATAC-seq data of all the ES and LS samples. **(C)** The STRING network analysis of pathways enriched for ES-specific ATAC-seq peak-associated genes.

Supplementary Figure S14 Integrative analysis of ATAC-seq peaks with copy number variation. **(A)** Boxplot shows the relationship between copy number (normal, gain and loss) and the signal intensity of ATAC-seq peak that located in corresponding regions. **(B)** Scatterplot represents the correlation between the copy number of CNV gain regions and the signal intensity of ATAC-seq peaks.

Supplementary Figure S15 KEGG pathway enrichment analysis. ES-specific ATAC-seq peak-associated genes **(A)** and LS-specific ATAC-seq peak-associated genes **(B)**.
