# Supplementary figures and images for: Integrated Multiomics Analyses Revealing Different Molecular Profiles Between Early- and Late-Stage Lung Adenocarcinoma
Source: Front Oncol. 2021 Oct 21;11:746943. doi: 10.3389/fonc.2021.746943 (PMC8567144; doi:10.3389/fonc.2021.746943)

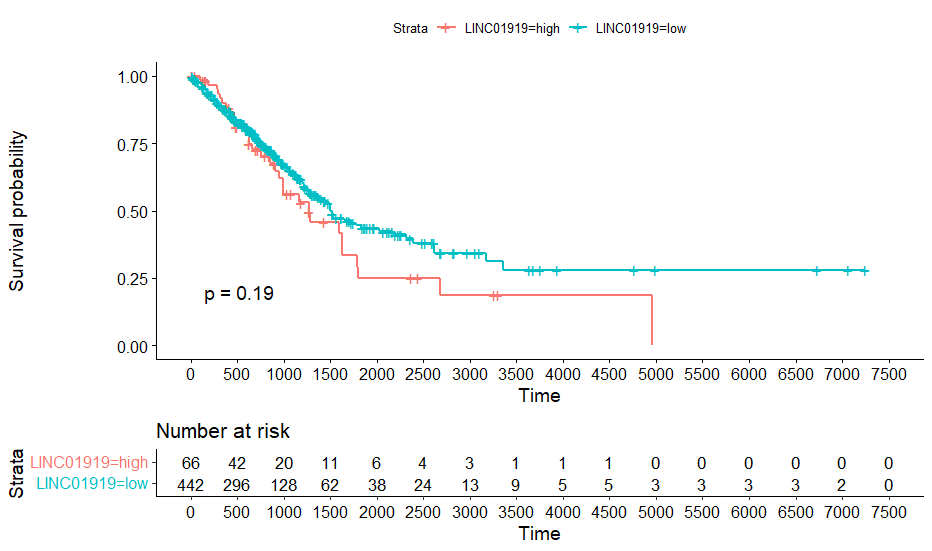

Supplement: Supplementary Figure S1 — Representative HE staining images of ES and LS tissues (×4 magnification; the lower right corner, ×40 magnification). [file DataSheet_1.zip › Supplementary Figure S12-version2.tif]

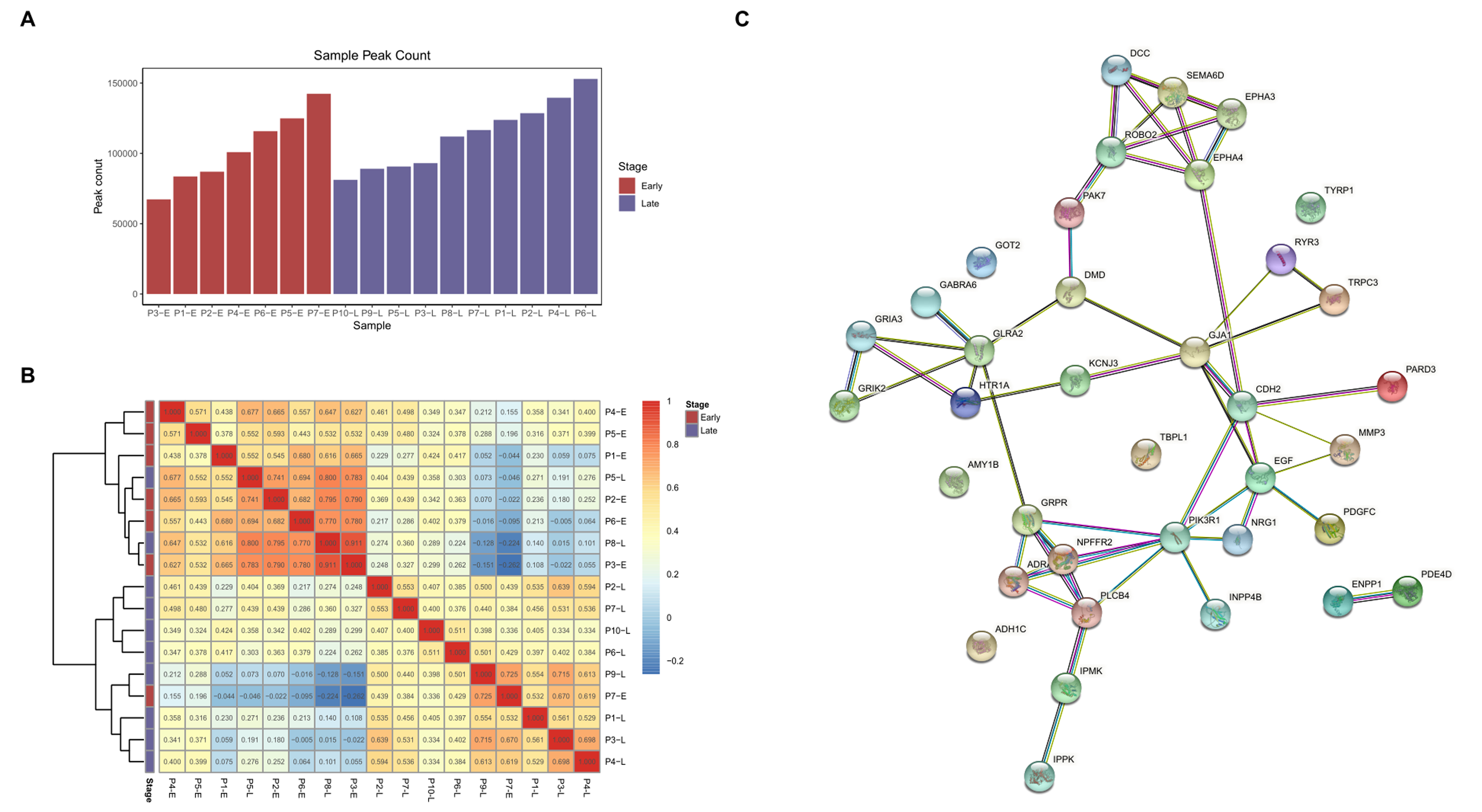

Supplement: Supplementary Figure S1 — Representative HE staining images of ES and LS tissues (×4 magnification; the lower right corner, ×40 magnification). [file DataSheet_1.zip › Supplementary Figure S13-version2.tif]

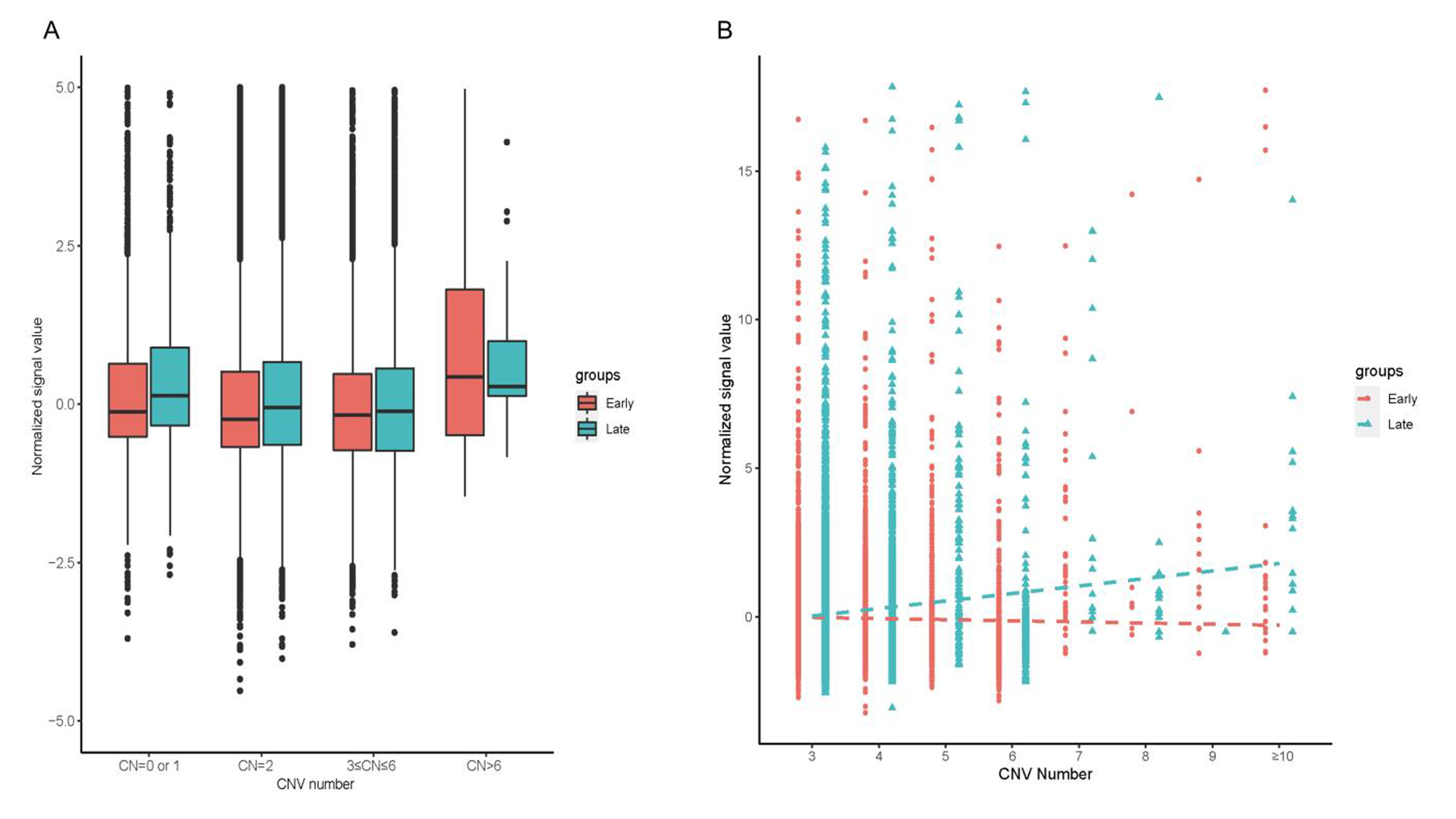

Supplement: Supplementary Figure S1 — Representative HE staining images of ES and LS tissues (×4 magnification; the lower right corner, ×40 magnification). [file DataSheet_1.zip › Supplementary Figure S14-version2.tif]

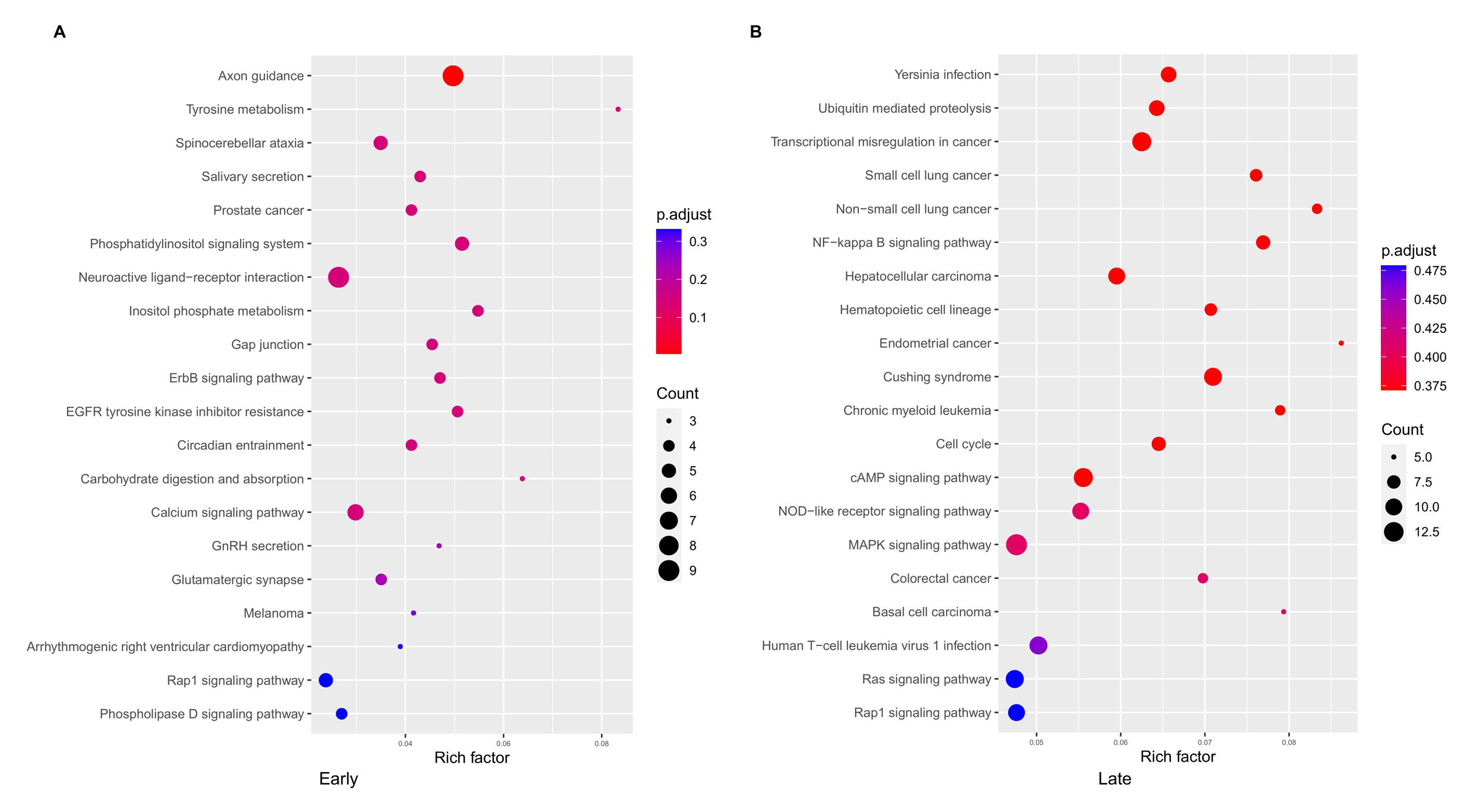

Supplement: Supplementary Figure S1 — Representative HE staining images of ES and LS tissues (×4 magnification; the lower right corner, ×40 magnification). [file DataSheet_1.zip › Supplementary Figure S15-version2.tif]

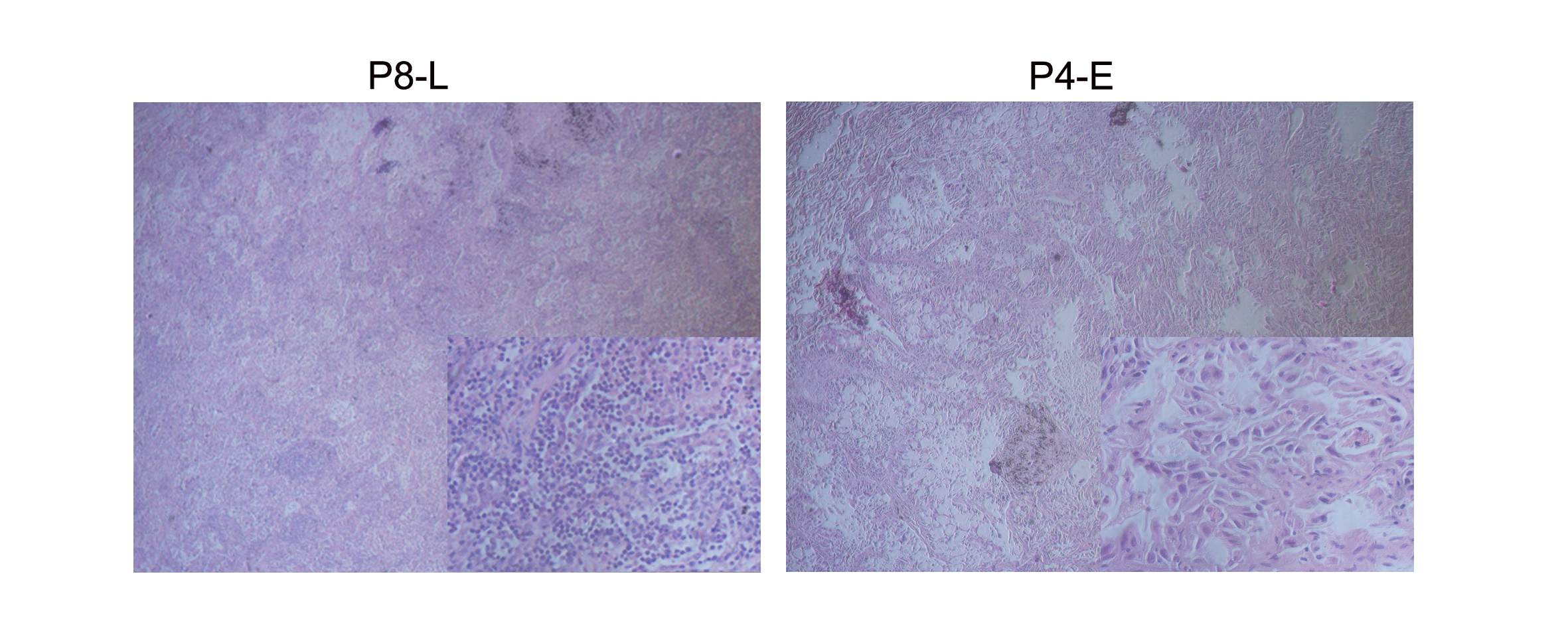

Supplement: Supplementary Figure S1 — Representative HE staining images of ES and LS tissues (×4 magnification; the lower right corner, ×40 magnification). [file DataSheet_1.zip › Supplementary Figure S1-version2.tif]

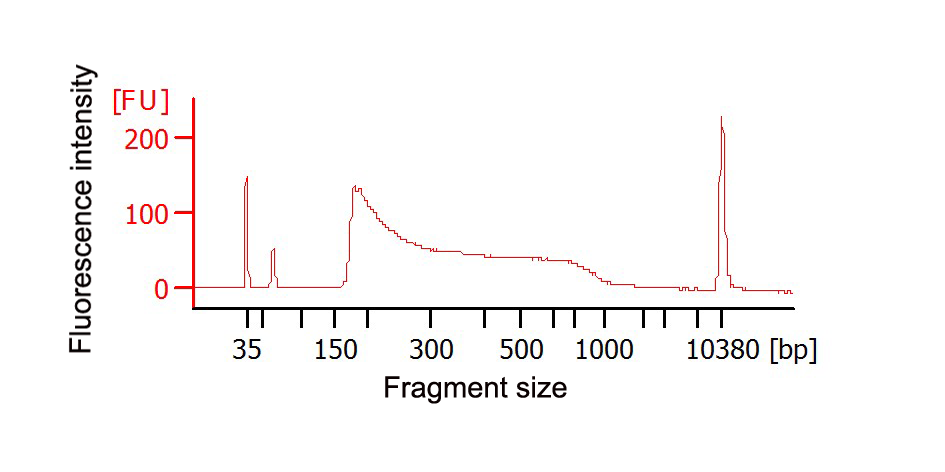

Supplement: Supplementary Figure S1 — Representative HE staining images of ES and LS tissues (×4 magnification; the lower right corner, ×40 magnification). [file DataSheet_1.zip › Supplementary Figure S2--version2.tif]

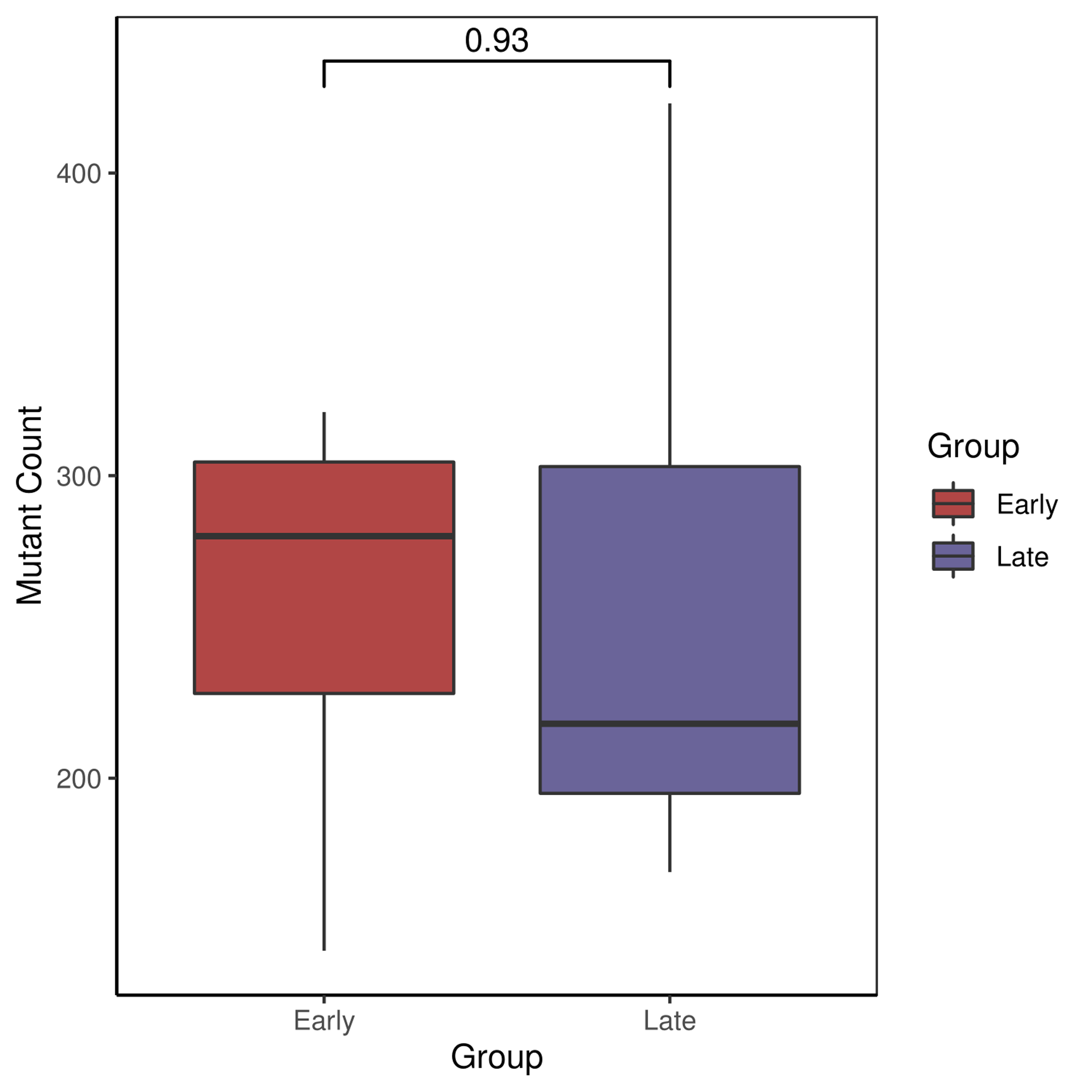

Supplement: Supplementary Figure S1 — Representative HE staining images of ES and LS tissues (×4 magnification; the lower right corner, ×40 magnification). [file DataSheet_1.zip › Supplementary Figure S3-version2.tif]

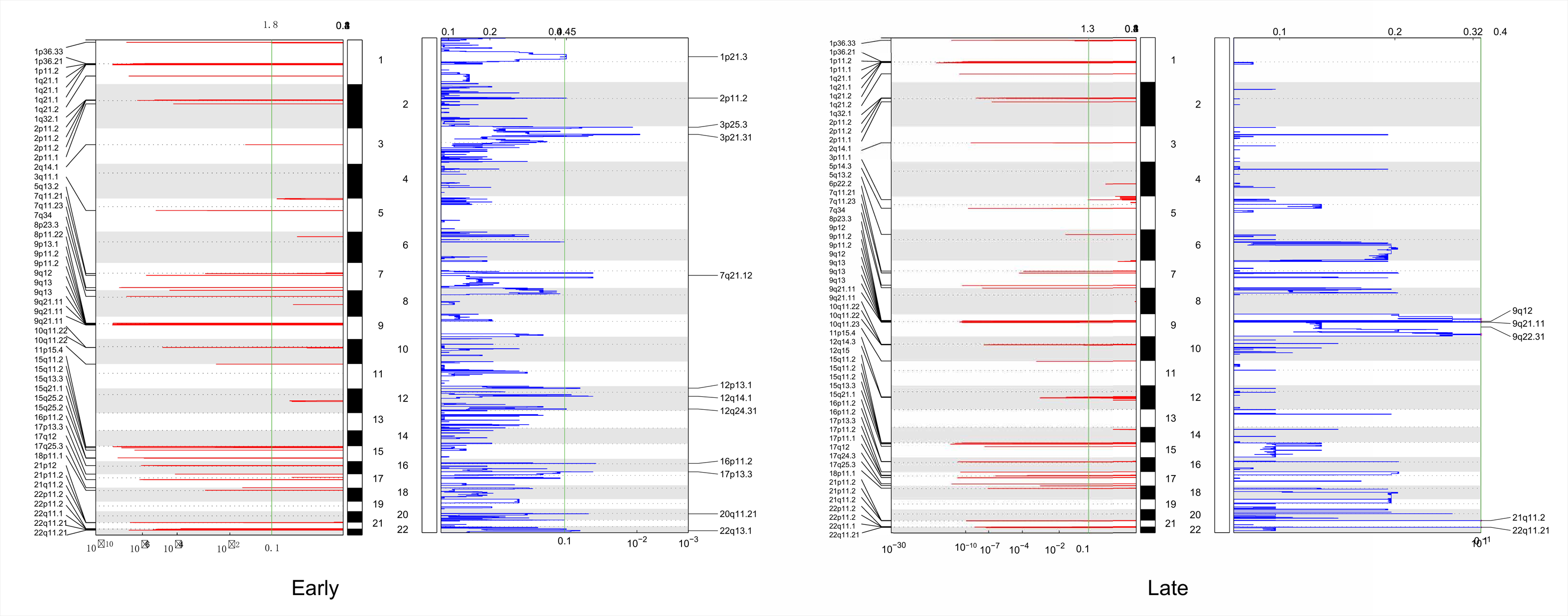

Supplement: Supplementary Figure S1 — Representative HE staining images of ES and LS tissues (×4 magnification; the lower right corner, ×40 magnification). [file DataSheet_1.zip › Supplementary Figure S4-version2.tif]

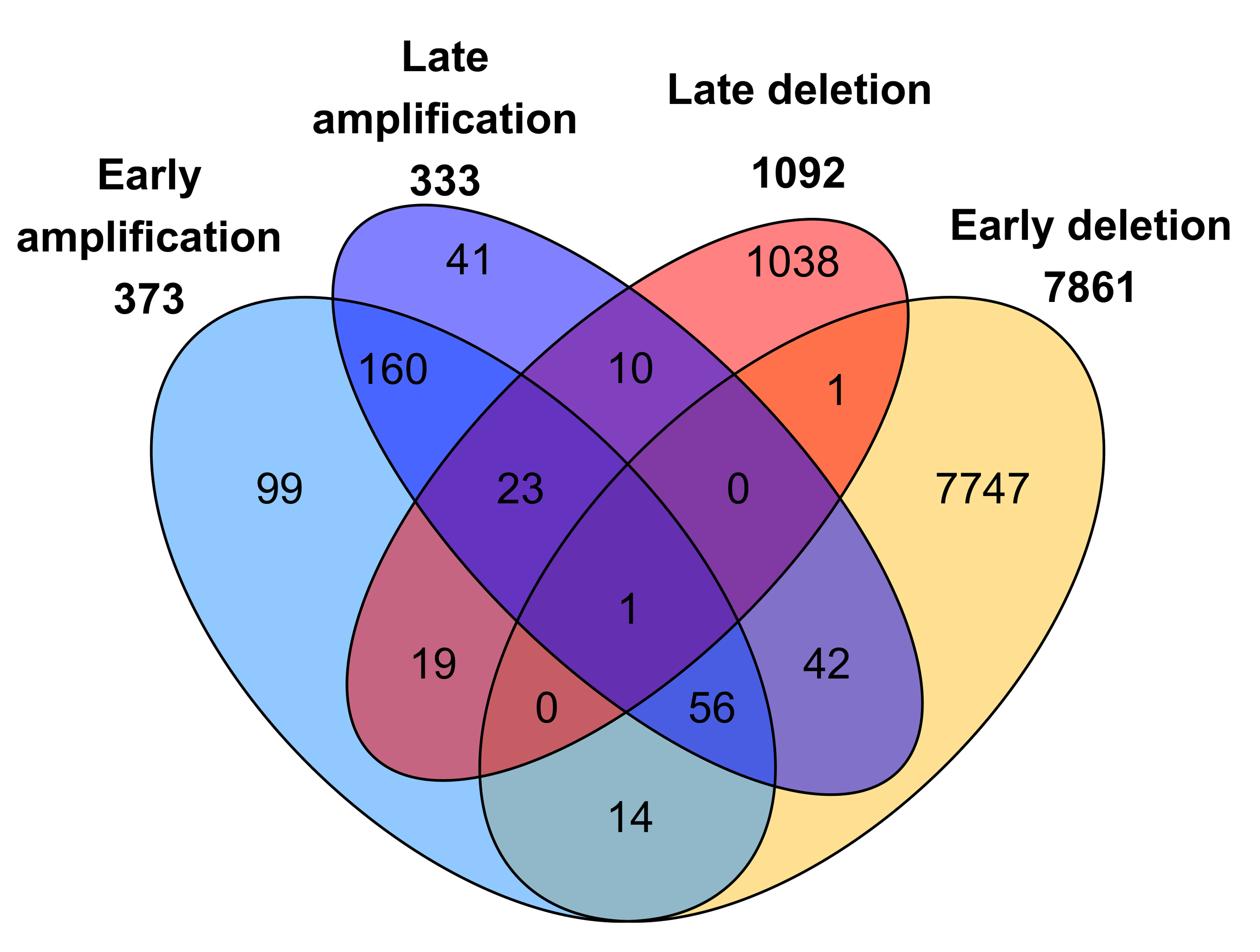

Supplement: Supplementary Figure S1 — Representative HE staining images of ES and LS tissues (×4 magnification; the lower right corner, ×40 magnification). [file DataSheet_1.zip › Supplementary Figure S5-version2.tif]

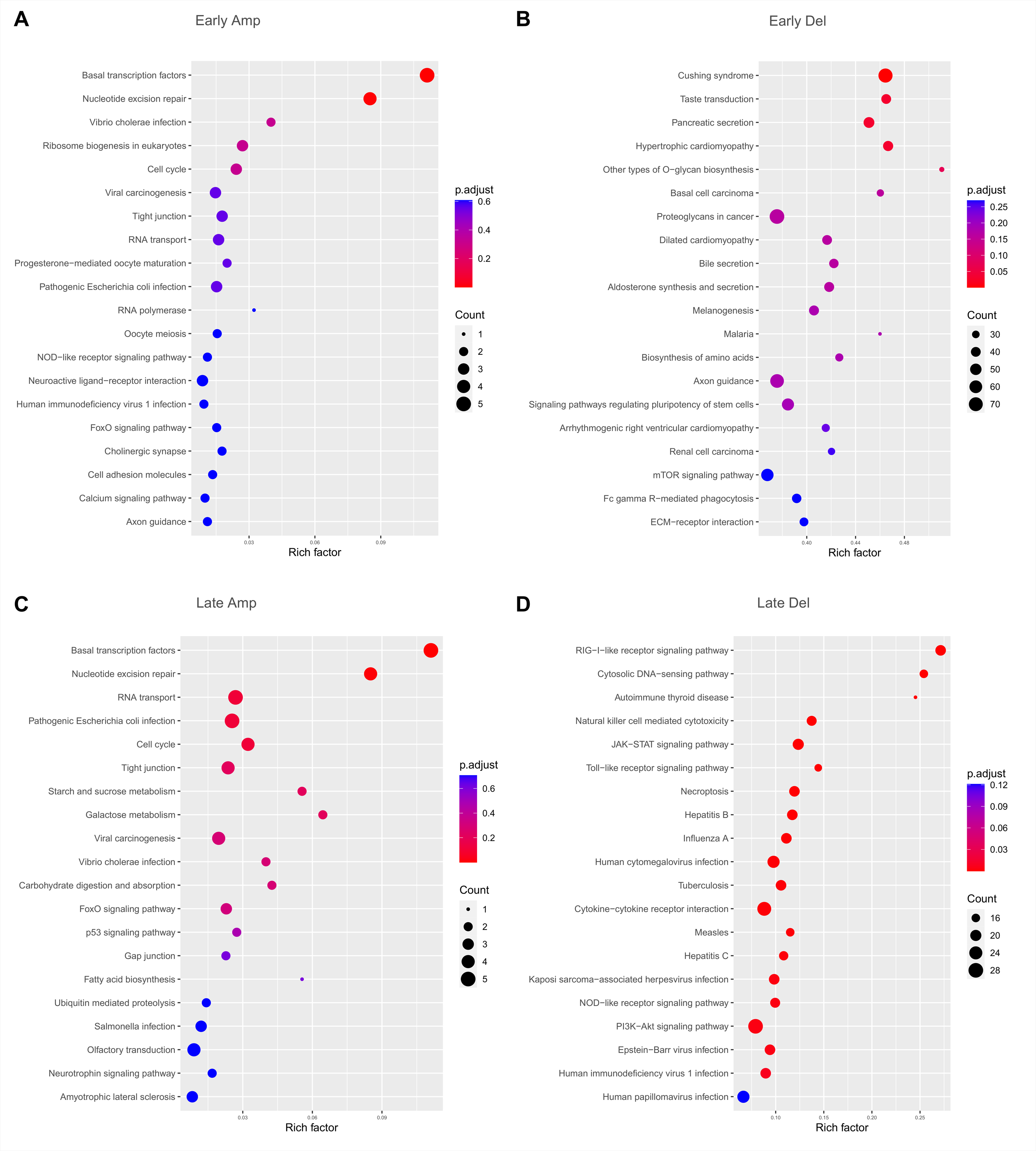

Supplement: Supplementary Figure S1 — Representative HE staining images of ES and LS tissues (×4 magnification; the lower right corner, ×40 magnification). [file DataSheet_1.zip › Supplementary Figure S6-version2.tif]

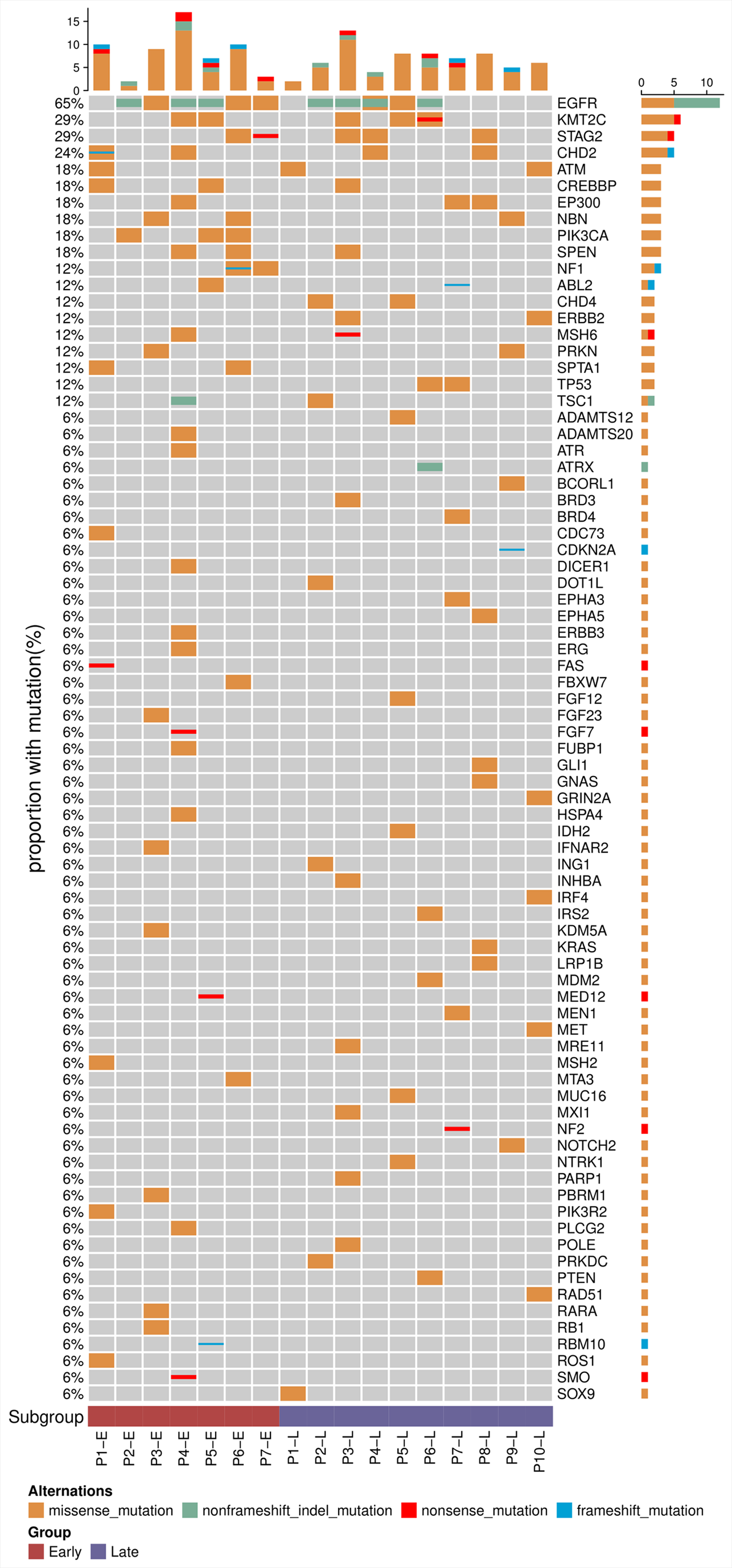

Supplement: Supplementary Figure S1 — Representative HE staining images of ES and LS tissues (×4 magnification; the lower right corner, ×40 magnification). [file DataSheet_1.zip › Supplementary Figure S7-version2.tif]

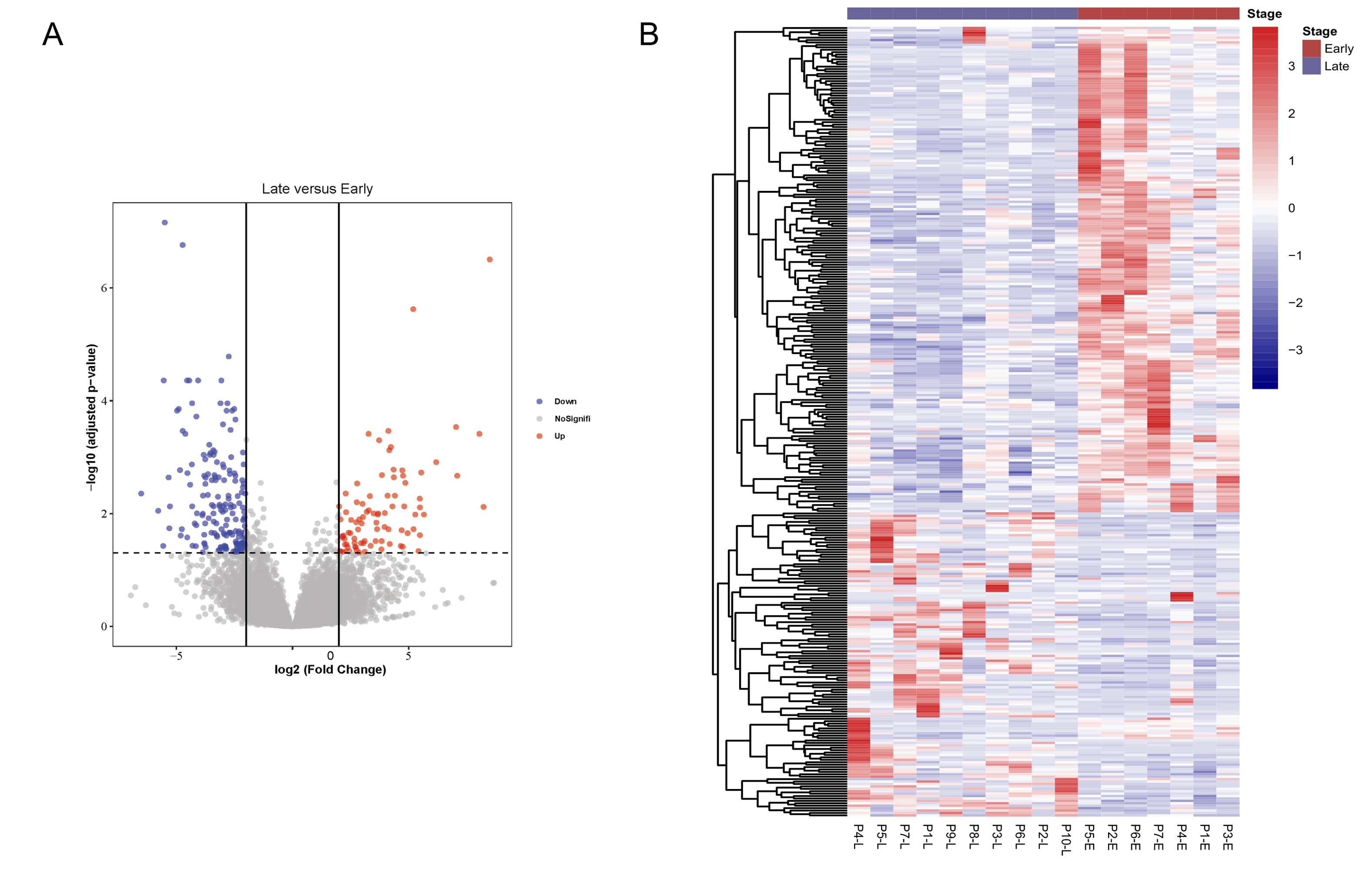

Supplement: Supplementary Figure S1 — Representative HE staining images of ES and LS tissues (×4 magnification; the lower right corner, ×40 magnification). [file DataSheet_1.zip › Supplementary Figure S8-version2.tif]
