# Supplementary material for: Integrated Multiomics Analyses Revealing Different Molecular Profiles Between Early- and Late-Stage Lung Adenocarcinoma
Source: Front Oncol. 2021 Oct 21;11:746943. doi: 10.3389/fonc.2021.746943 (PMC8567144; doi:10.3389/fonc.2021.746943)
Supplement: Supplementary file 2 [file DataSheet_2.zip › Supplementary Table S2.docx]

**Supplementary Table S2. Clinical information of early and late stage of LUAD patients.**

| **Patient No.** | **Age** | **Gender** | **Smoking history** | **Stage** | **Histological type** |
| --- | --- | --- | --- | --- | --- |
| P1-E | 59 | Female | Non-smoking | IA | Adenocarcinoma |
| P2-E | 66 | Male | Non-smoking | IA | Adenocarcinoma |
| P3-E | 59 | Male | Non-smoking | IA | Adenocarcinoma |
| P4-E | 67 | Male | Non-smoking | IA | Adenocarcinoma |
| P5-E | 51 | Male | Non-smoking | IA | Adenocarcinoma |
| P6-E | 48 | Female | Non-smoking | IA | Adenocarcinoma |
| P7-E | 64 | Female | Non-smoking | IA | Adenocarcinoma |
| P1-L | 56 | Female | Non-smoking | IIIB | Adenocarcinoma |
| P2-L | 46 | Male | Non-smoking | IIIB | Adenocarcinoma |
| P3-L | 50 | Male | Non-smoking | IV | Adenocarcinoma |
| P4-L | 52 | Male | Non-smoking | IV | Adenocarcinoma |
| P5-L | 56 | Female | Non-smoking | IV | Adenocarcinoma |
| P6-L | 48 | Female | Non-smoking | IV | Adenocarcinoma |
| P7-L | 33 | Male | Non-smoking | IV | Adenocarcinoma |
| P8-L | 49 | Male | Non-smoking | IIIB | Adenocarcinoma |
| P9-L | 61 | Female | Non-smoking | IIIA | Adenocarcinoma |
| P10-L | 62 | Female | Non-smoking | IIIA | Adenocarcinoma |
