# Supplementary material for: Integrated Multiomics Analyses Revealing Different Molecular Profiles Between Early- and Late-Stage Lung Adenocarcinoma
Source: Front Oncol. 2021 Oct 21;11:746943. doi: 10.3389/fonc.2021.746943 (PMC8567144; doi:10.3389/fonc.2021.746943)
Supplement: Supplementary file 2 [file DataSheet_2.zip › Supplementary Table S4.doc]

**Supplementary Table S4. Incidence of mutations by gene in patients with early stage and late stage lung adenocarcinoma in the cBioPortal dataset**

| **Gene** | **I**  **(n=90)** | **Ⅱ**  **(n=36)** | **Ⅲ**  **(n=22)** | **Ⅳ**  **(n=10)** | **ES**  **(I)**  **(n=90)** | **LS (III+IV)**  **(n=32)** |
| --- | --- | --- | --- | --- | --- | --- |
| PIK3CA | 4 | 2 | 2 | 0 | 4.4% | 6.3% |
| NF1 | 6 | 7 | 0 | 2 | 6.7% | 6.3% |
| CHD4 | 2 | 0 | 0 | 1 | 2.2% | 3.1% |
| ERBB2 | 1 | 1 | 1 | 1 | 1.1% | 6.3% |
| TP53 | 42 | 20 | 11 | 5 | 46.7% | 50.0% |
